# The Potential Role of Regulatory B Cells in Idiopathic Membranous Nephropathy

**DOI:** 10.1155/2020/7638365

**Published:** 2020-12-21

**Authors:** Zhaocheng Dong, Zhiyuan Liu, Haoran Dai, Wenbin Liu, Zhendong Feng, Qihan Zhao, Yu Gao, Fei Liu, Na Zhang, Xuan Dong, Xiaoshan Zhou, Jieli Du, Guangrui Huang, Xuefei Tian, Baoli Liu

**Affiliations:** ^1^Beijing University of Chinese Medicine, No. 11, North Third Ring Road, Chaoyang District, Beijing 100029, China; ^2^Beijing Hospital of Traditional Chinese Medicine Affiliated to Capital Medical University, No. 23 Meishuguanhou Street, Dongcheng District, Beijing 100010, China; ^3^Shandong First Medical University, No. 619 Changcheng Road, Tai'an City, Shandong 271016, China; ^4^Shunyi Branch, Beijing Traditional Chinese Medicine Hospital, Station East 5, Shunyi District, Beijing 101300, China; ^5^Beijing Chinese Medicine Hospital Pinggu Hospital, No. 6, Pingxiang Road, Pinggu District, Beijing 101200, China; ^6^Capital Medical University, No. 10, Xitoutiao, You'anmenwai, Fengtai District, Beijing 100069, China; ^7^Department of Internal Medicine, Yale University School of Medicine, New Haven, Connecticut, USA

## Abstract

Regulatory B cells (Breg) are widely regarded as immunomodulatory cells which play an immunosuppressive role. Breg inhibits pathological autoimmune response by secreting interleukin-10 (IL-10), transforming growth factor-*β* (TGF-*β*), and adenosine and through other ways to prevent T cells and other immune cells from expanding. Recent studies have shown that different inflammatory environments induce different types of Breg cells, and these different Breg cells have different functions. For example, Br1 cells can secrete IgG4 to block autoantigens. Idiopathic membranous nephropathy (IMN) is an autoimmune disease in which the humoral immune response is dominant and the cellular immune response is impaired. However, only a handful of studies have been done on the role of Bregs in this regard. In this review, we provide a brief overview of the types and functions of Breg found in human body, as well as the abnormal pathological and immunological phenomena in IMN, and propose the hypothesis that Breg is activated in IMN patients and the proportion of Br1 can be increased. Our review aims at highlighting the correlation between Breg and IMN and proposes potential mechanisms, which can provide a new direction for the discovery of the pathogenesis of IMN, thus providing a new strategy for the prevention and early treatment of IMN.

## 1. Introduction

The human immune system has the ability to distinguish self and nonself. Under normal circumstances, the immune system only produces an immune response to nonautoantigens, while it has no response to the autoantibodies or only produces a weak response; that is, the immune system is in an immune tolerance state to its own tissue components [1, 2]. In the state of immune tolerance, there are certain amounts of autoantibodies against autoantigens or autoreactive T cells and autoreactive B cells, thus producing an autoimmune response. This response is physiological, and its main function is to clear the aging in vivo apoptotic or aberrant autocells [3–5]. However, when the autoimmune tolerance state is broken, the immune system produces a pathological immune response to the autoantigen, resulting in the damage or dysfunction of its own tissues and cells, thus forming an autoimmune disease [6, 7]. The characteristics of autoimmune diseases compared with other diseases is that the patients can be detected with autoreactive T and B cells which can cause immune damage to the components of their own tissues [8, 9]. B cells play a crucial role in autoimmune diseases in particular.

B cells are professional antigen-presenting cells (APCs) and can differentiate into plasma cells capable of producing autoantibodies [8, 10, 11]. B cells are both the beginning of autoimmune diseases and the effect of most autoimmune diseases. Breg, as one of the members of B cells, plays a vital role in immune regulation, exerting a myriad of influences on the occurrence and development of autoimmune diseases, such as type I diabetes, lupus erythematosus, and rheumatoid arthritis [11–15].

IMN belongs to autoimmune nephropathy and is one of the common pathological types of nephrotic syndrome [16]. The increase in the incidence of this disease and the discovery of autoantigens such as phospholipase A2 receptor (PLA2R) attracted the attention of many researchers [17]. Although few basic studies have been published on IMN and its immunological mechanisms, the efficacy of rituximab has encouraged researchers to focus on B-cell studies [18, 19]. At the same time, the oligoinflammatory nature of IMN makes it different from other autoimmune nephropathies, which indicates the important role of immune regulation in the pathogenesis of this disease [11]. Therefore, we reviewed the role of B cells in autoimmune diseases and attempted to hypothesize the relationship between Bregs and IMN.

## 2. Overview of Breg

### 2.1. A Brief History of Breg

The activation of the immune system is compulsory. However, if the occurrence of autoimmune reactions such as autoimmunity against autoantigens, immunity against allogeneic fetus, hypersensitivity against allergens, and immune response against normal intestinal flora is not effectively suppressed, it will lead to serious autoimmune diseases [20–22]. Therefore, the weakening of the immune response is more important. Besides being able to recognize antigens such as APC, B cells can be differentiated into plasma cells to secrete antibodies and provide immune protection for the human body [11]. It is important to understand how B cells properly control the immune response under normal conditions and suppress a wrong or excessive immune response to avoid damage to the body. This part of the function of controlling the uncontrolled immune response belongs to immune regulation, and the B cells responsible for immune regulation become Bregs [23].

The discovery of Bregs is almost parallel to that of regulatory T cells (Tregs), but less importance was given to Breg. As early as 1974, Neta and Salvin discovered a B cell in the spleen of guinea pigs that specifically inhibits delayed hypersensitivity [24]. In 1996, Janeway et al. found that B-cell-deficient mice were allergic to myelin oligodendrocyte protein (MOG) and were unable to prevent the occurrence of experimental autoimmune encephalomyelitis (EAE) that worsened the autoimmune response [25]. These studies have shown that there is the presence of a type of B cell involved in immune regulation. In 2002, Fillatreau et al. found that these B cells secrete IL-10 [26]. In 2006, the concept of Breg was formally proposed by Mizoguchi and Bhan [27]. Since then, there have been numerous studies on Breg, mostly in mice; however, the existence of Breg in humans remains a mystery. In 2010, Blair et al. found the presence of Breg in lupus patients [28]. In 2011, Iwata et al. found the presence of Breg in human peripheral blood performing the same function as B10 cells in mice [29]. B10 cells are a subtype of mouse Breg. The phenotype of this cell is CD5^+^CD1d^hi^, while in humans this phenotype is CD24^hi^CD27^+^, which suggests that Breg phenotypes in humans are not universal in relation to those derived from animal studies. Thus, the academic world began a new era of exploration of Breg species and their functions in the human body.

### 2.2. Types and Functions of Breg

**Figure 1:**
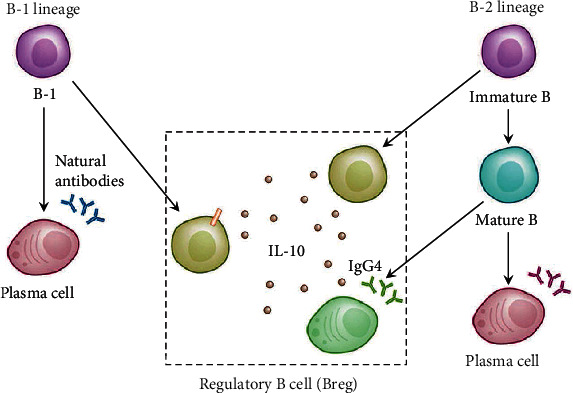
The source of Breg. Different from Treg, Breg has more extensive sources. It does not matter whether they are B1 cells or B2 cells, or if they are immature B cells or mature B cells, under certain conditions of stimulation, Breg with immunomodulation function can be differentiated. At present, the main mechanism by which Breg plays the immune regulatory function in the human body is the secretion of IL-10.

Breg production differs from Tregs, i.e., it can be differentiated from B cells at different stages and show the functions of B cells at different stages (Figure 1) [23]. This also leads to a wide variety of Breg phenotypes. Different phenotypes of human Bregs are known in Table 1. Different types of Bregs play different functions and regulate different autoimmune responses to different immune cells.

#### 2.2.1. The Role of IL-10 in Breg Function

**Figure 2:**
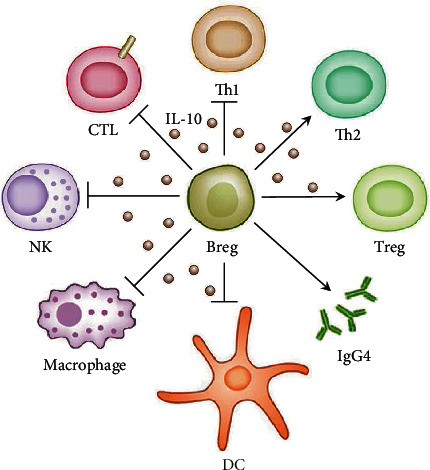
The function of Breg. Breg can promote Th2 cell differentiation and inhibit Th1 cell differentiation by secreting IL-10. In addition, its secretion of inhibitory cytokines such as IL-10, TGF-*β*, and adenosine can inhibit the activity of NK cells, monocytes, and CTL, and impair the antigen presentation function of dendritic cells. In addition, some Bregs can also promote the differentiation of Treg, enhance the immune regulation effect, and secrete IgG4 blocking autoantibodies to protect the body from hypersensitivity damage.

Breg plays an immunosuppressive role in most people mainly by secreting IL-10 (Figure 2) [28–34]. It can also suppress the function of different kinds of immune cells. In terms of T cells, Breg inhibited the production of interferon-*γ* (IFN-*γ*) and IL-17 and other proinflammatory cytokines by Th1 and Th17 through IL-10, and inhibited the differentiation of initial T cells into Th1 and Th17 [35–38]. IL-10 can also directly inhibit the activity of cytotoxic T lymphocytes (CTL) and ultimately inhibit cellular immunity [39, 40]. In addition, in the IL-10 environment, Breg expressing B7 can induce the differentiation of Treg and Tr1 and thus promote the occurrence of immune regulation; however, not all Breg can do this [41]. Therefore, Breg plays a key role in immune regulation through the dynamic diversification interaction between secreted IL-10 and T cells. In addition to T cells, Breg can inhibit the expression of major histocompatibility complex II (MHC II) on the surface of dendritic cells by secreting IL-10, thereby inhibiting antigen presentation [42–44]. In addition, IL-10 can also inhibit the activity of monocytes and natural killer cells (NK cells), thereby inhibiting the innate immune response [45–47]. It is worth noting that IL-10 promotes the proliferation of B cells, which may lead to the possibility that Breg can play an accessory role in regulating adaptive immunity in the continuation of humoral immune response [46].

#### 2.2.2. In Addition to IL-10 in Breg Function

In addition to the secretion of IL-10, Breg can achieve immunomodulatory effects through other means. Tim-1 B cells, as a type of Breg, can secrete not only IL-10 but also TGF-*β* to play an immunomodulation role [31]. TGF-*β* can inhibit the proliferation and differentiation of various immune cells and the production of cytokines, especially regulating the differentiation of Th17 and Treg cells [31, 48, 49]. It also promotes the proliferation of fibroblasts, osteoblasts, and Schwann cells to help repair damaged tissues [50–53]. In addition, Th9 differentiation requires TGF-*β* and IL-4 induction, and some allergic inflammation is associated with Th9 overactivation [54, 55]. It is worth noting that Breg which can secrete IL-35 has not been found in the human body, and the specific mechanism needs to be explored [56, 57]. In addition to promoting Treg differentiation, immature B cells with immunomodulation can express programmed cell death protein ligand 1 (PD-L1), which can directly kill T cells [58]. Recent studies have shown that in terms of infection, Breg in human immunodeficiency virus (HIV) patients inhibited antigen presentation and the activity of CD4^+^ T cells through interaction between IL-10 and PD-1/PD-L1, and it inhibited anti-HIV cytotoxic T lymphocytes at the same time [59]. In terms of tumor, PD-L1 is highly expressed in tumor invasive B cells in patients, which may assist the tumor to achieve immune escape [60]. At the same time, the presence of B cells with a high expression of PD-L1 in malignant B lymphoblastoma provides a theoretical basis for PD-1 inhibitor treatment of this disease [61, 62]. In addition to secreting IL-10, GrB^+^ B cells also play an immunosuppressive role by producing granzyme B (GrB) and indoleamine 2,3-dioxygenase (IDO) [30]. The phenotype of this cell is CD38^+^CD1d^+^IgM^+^CD147^+^. GrB^+^ B cells can infiltrate tumors and inhibit the CD4^+^ T cell response after being activated by IL-21 [63]. Breg with phenotype CD39^+^CD73^+^ can secrete adenosine, which affects the activity and proliferation of various immune cells, mainly neutrophils, monocytes, and T cells, and plays an immunomodulatory function [64]. In vitro, such cells can be activated in IL-4 by stimulating their CD40 [64]. Breg with IgD^lo^CD38^+^CD24^lo^CD27^−^ controls B7 expression through CD62L, thereby inhibiting dendritic cell maturation [65]. Br1 cells with phenotype CD25^hi^CD71^hi^ can directly secrete IgG4 to block autoantigens, and this antibody is currently considered to have a protective effect in patients with chronic allergy [33]. We can activate this type of cell by stimulating its TLR9 [33]. To sum up, Breg mainly inhibits innate immune response and cellular immune response in specific immunity, but it lacks corresponding means to regulate humoral immune response.

## 3. Abnormal Features of IMN

### 3.1. Abnormal Pathological Features of IMN

IMN is one of the common pathologic types of nephrotic syndrome. At an early stage, the glomerular subepithelial deposits can only be seen under a light microscope, and then the glomerular basement membrane is diffusely thickened [16]. At late stages, the mesangial matrix increases, capillary loops are compressed and occluded, and glomerulosclerosis occurs [66]. However, the glomerulus is usually free of cell proliferation and immune cell infiltration throughout the process [67]. In immunofluorescence examination, IgG was diffusely deposited in the glomerular capillary wall, but not in IgM [68]. Most patients are associated with C3 deposition [69]. In general, there are no deposits of multiple immunoglobulin and complement C1q, and they are not deposited in the area outside the glomerular capillary wall. With the detection of the anti-PLA2R antibody on serum and kidney sections, IgG deposited on the glomerular capillary wall of IMN patients is the most common IgG, followed by IgG1 [68].

It is not difficult to see that the pathological characteristics of IMN are very abnormal. Firstly, the antigen antibody complex was deposited only under the glomerular epithelial cells, and mesangial cell proliferation was rare, indicating that the immune complex was generated by an in situ immune complex [70, 71]. Recently, Liu et al. hypothesized that the autoantigen of IMN came from human lungs [72]. This statement broke the current research deadlock and was widely recognized by peers. Secondly, IgG4 is the dominant antibody in patients, indicating that the disease is at the end of the immune response [73–75]. The pathogenesis of IMN is necessarily different from that of IgG4-related diseases (IgG4-RD), because IMN does not have systemic multitissue IgG4^+^ plasma cell infiltration like IgG4-RD, and the incidence of kidney disease is concentrated in the renal interstitium [76, 77]. Thirdly, although IgG and C3 deposition can be seen in the renal tissues of IMN patients, C1q deposition is rare, and there is no obvious abnormality in serum C1q and C3, indicating that there is no significant complement depletion in the patients [78, 79]. Also, the passive Heymann nephritis rat model has demonstrated that C5b-9 deposition is one of the factors leading to proteinuria, and the complement of IMN patients can be activated by the alternative pathway or the mannose-binding lectin pathway [79–82]. However, there are no reports on the effectiveness of the direct use of eculizumab in the treatment of this disease, which indicates that kidney damage in IMN patients is not mainly caused by the complement [83]. Many contradictory phenomena have obscured the pathogenesis of IMN. So let us see what happens to the immune cells and cytokines in this disease.

### 3.2. Abnormal Immunological Characteristics of IMN

As an autoimmune disease, the main autoantibodies of IMN are PLA2R, accounting for 70-80% [84–86]. PLA2R is a member of the C-type lectin superfamily, also known as the mannose receptor family [87–89]. It is a type I transmembrane protein that contains long extracellular segments, long transmembrane segments, and short intracellular segments [88]. It has been reported that PLA2R inhibits inflammatory response by binding to the PLA2 protein, and the increased expression of PLA2R is related to inflammatory stimulation and aging [72, 90, 91]. This also proves that IMN is more common among the elderly. The onset of this disease is related to environmental pollution or the history of respiratory tract infection during the adolescent period [92, 93]. At the gene level, Stanescu et al. found that the HLA-DQA1 allele on chromosome 6p21 was closely related to the pathogenesis of IMN after collecting the relevant data of 556 white IMN patients [94]. The single nucleotide polymorphism of PLA2R and HLA-DQA1 can be related to IMN susceptibility [94, 95]. Interestingly, about 7% of patients with this disease were seropositive for the anti-PLA2R antibody but negative for renal tissue PLA2R [96]. There are also many cases of recurrence of IMN after renal transplantation [97]. However, this protein is expressed in the lungs and kidneys [82, 98], and it is also found in neutrophils and alveolar macrophages [99–101]. The evidence raises the question of whether IMN's autoimmune response really originated in the kidney.

In terms of immune cells, there was no significant increase in the number of T and B cells in the patients [102, 103]. In terms of T cells, the most significant reduction was in CD8^+^ T cells, while Th2 cells accounted for the largest proportion in CD4^+^ T cells, and the number of Th1 and Treg cells decreased [103, 104]. In terms of cytokines, IL-4, IL-10, and IL-13 were significantly increased, while there was no significant change in IFN-*γ* and IL-12 [103, 105–107]. Ifuku et al. compared the expression levels of renal cytokine mRNA with those of antineutrophil cytoplasmic autoantibody-associated crescentic glomerulonephritis (ANCAGN) and membrane proliferative glomerulonephritis (MPGN) in IMN patients and found that IL-6, IL-12, and IL-17 in IMN patients were significantly reduced compared with the other two groups, while IL-4, IL-5, TGF-*β*, and Foxp3 significantly increased compared with the other two groups [108]. Kawasaki et al. compared the serum cytokine levels of IMN children with lupus nephritis children, MPGN, Henoch-Schönlein purpura nephritis, and IgA nephritis, and found that serum IL-2, IL-6, IL-12, and IFN-*γ* were not significantly increased, while the contents of IL-4 were significantly increased [109]. All these cytokines with insufficient content have the effect of strengthening cellular immune response, while the increased cytokines have the effect of strengthening humoral immune response and inhibiting cellular immunity. From the perspective of source cells, IL-6 is mainly produced by mononuclear macrophages, endothelial cells, fibroblasts, and other cells [110–112]. IL-12 is mainly produced by dendritic cells, macrophages, and B cells [113, 114]. IFN-*γ* is mostly produced by NK cells and Th1 cells [115, 116]. Therefore, IMN is a disease dominated by humoral immune response, while the innate immune response and cellular immune response in specific immunity are weakened.

## 4. Role of Breg in IMN

### 4.1. Breg Activation Is Present in IMN

Professional APCs recognize their own antigens, activate T helper cells, and induce activation of cellular and humoral immunity. The antibodies produced in the first response of humoral immunity are mainly IgM, and IgG can be produced at a later period [117, 118]. Although cytokines produced by activation of humoral immune response, such as IL-4 and IL-10, can weaken cellular immune response [119, 120], cellular immune response of a large number of autoimmune diseases coexists with humoral immune response [121–124], indicating that cellular immunity will not be easily attenuated due to activation of humoral immunity. However, when the immunomodulatory cells intervene, the innate and specific immune responses are weakened, and the humoral immune response is the main stream. This is precisely because whether Treg or Breg play an immunomulatory role, they mainly rely on the secretion of IL-10, IL-35, and TGF-*β* to suppress cellular immunity, but lack the means to secrete IFN-*γ* to inhibit Th2 differentiation or block B cell-activating factor receptor (BAFF-R), B cell maturation antigen (BCMA), transmembrane activator, and CAML interactor (TACI), and other ways to inhibit B cell proliferation, thus inhibiting humoral immune response [125–129]. Therefore, for IMN, where humoral immune response is dominant, only active immune regulation can explain all kinds of abnormalities in IMN. The presence of tumor-related MN, and the pathological characteristics of the disease are very similar to those of IMN, is a strong evidence of active immune regulation [85, 130, 131]. Tumors are known to escape the body's immune system by escaping [132, 133]. Autoimmune diseases, however, are characterized by overactivation of the immune system. Therefore, if the cells play an immunomodulatory role, they can participate in and even lead to the occurrence of autoimmune diseases. In this environment, tumors can coexist with autoimmune diseases [134, 135].

Interestingly, however, the peripheral number of Treg and the blood IL-35 content of IMN patients were lower than those of normal people, indicating that Treg activity in IMN patients was insufficient [104, 136]. As mentioned above, Breg which can secrete IL-35 has not been found in humans. Therefore, Breg should be the main immune regulatory cells in IMN patients [56, 137]. Moreover, not all Bregs have the ability to activate Tregs. In addition, B cells themselves also belong to professional antigen-presenting cells [138, 139], although immune regulatory activation can lead to a decreased antigen presentation function of dendritic cells and macrophages, which are professional APCs [42–44]. Moreover, IMN patients contain more lipopolysaccharide (LPS) than normal people, which is enough to activate the resting B cells to perform an antigen presentation function [140, 141]. Meanwhile, B cells are mainly presented with soluble antigen, and the soluble PLA2R is present in the circulation of IMN patients [142, 143]. Hence, it is clear that under the activation of Breg, the autoimmune response can continue even if the antigen presentation ability of dendritic cells and macrophages is inhibited [42–44].

### 4.2. IgG4 Antibody Is Produced by Br1 Cells

**Figure 3:**
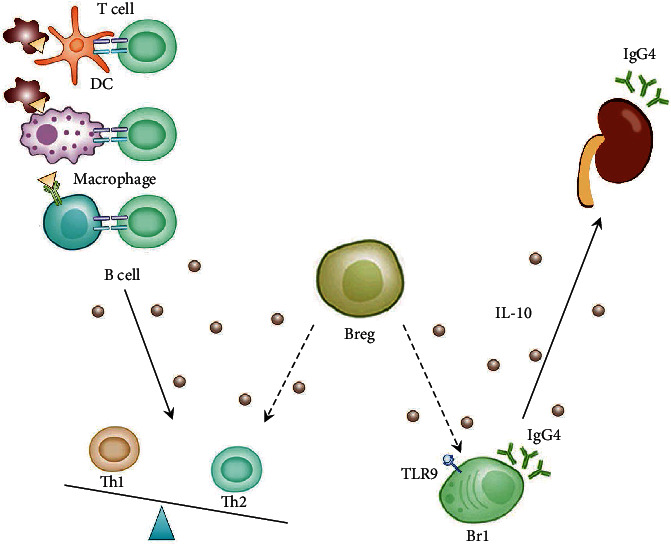
The role of Breg in IMN. Common autoantigens of IMN include PLA2R and THSD7A. Dendritic cells and macrophages phagocytose damaged cells or particles carrying this antigen, or B cells recognize the circulating soluble antigen. These professional APCs capture, process, and present their antigens to Th cells. Th cells reactivate B cells and induce differentiation. In an in vivo high-IL-10 environment, Br1 cells' TLR9 is also stimulated. This stimulates the secretion of IgG4 by Br1 cells. This happens even though IgG4 blocks autoantibodies, forming immune complexes beneath the glomerular epithelium, leading to IMN.

Br1 cells were discovered in 2013 by van de Veen et al. in beekeepers, people who had been chronically exposed to allergens, and in patients receiving desensitization therapy [33]. This cell production requires toll-like receptor 9 (TLR9) to be activated. The main means of immune regulation is secretion of IL-10 and production of the IgG4 antibody. However, the incidence of IMN is also related to air pollution; that is, long-term exposure of patients to air with excessive PM2.5 content meets the condition of long-term exposure to allergens [93]. In addition, the single nucleotide polymorphism of TLR9 is related to the pathogenesis of IMN [144]. Happily, Cantarelli et al. have confirmed the presence of increased Breg cells in the periphery of IMN patients [145]. The proliferation of these cells confirmed Br1. It is safe to assume that the patient has a factor that activates TLR9 or something that activates Br1. And our next study is to prove our point, in order to better study the pathogenesis of IMN (Figure 3).

### 4.3. Time Point of Breg Activation

To answer the time point of Breg activation in IMN progression, we should first determine when the cellular immune response in IMN patients is weaker than the cellular immune response. If it is in the course of disease, why is there no trace of cell infiltration found in the kidneys of a large number of IMN patients [146]? Namely, CD8^+^ T cell infiltration should be seen in the renal pathology of patients with early MN. If the PLA2R antigens were not from the kidney, IFN-*γ* concentrations or RNA levels in the peripheral blood should either be higher than at other time points or the proportion of CD8^+^ T cells should be higher [103, 134].

We do not deny the involvement of cellular immune response in IMN, but just why is this immune response so weak? However, in IMN patients, activation of the cellular immune response is inadequate at the onset of the disease. In other words, the level of IL-4 and IL-10 in IMN patients is already elevated before the onset of the disease, which can satisfy this phenomenon. This is because both of these cytokines are major cytokines that inhibit Th1 [36, 115, 147]. Among the cells that can secrete IL-4, basophils, NKT cells, and Th2 cells are common [148, 149]. Monocytes, mast cells, Th2 cells, Treg cells, and Breg cells are common among the cells that can secrete IL-10 [35, 36, 106, 150–153]. At present, there is no obvious correlation between mast cells and IMN [154]. Mononuclear cells showed no significant changes before and after treatment [106]. Treg cells showed low activity in IMN patients [104].

At the same time, no literature could prove the correlation between basophils and NKT cells and IMN. Therefore, Th2 and Breg levels were already elevated before the onset of the disease. To prove whether our opinion is valid, it only needs to prove that the IL-4 and IL-10 levels of patients before the diagnosis of IMN are higher than those of ordinary people, and the occurrence of hypoproteinemia and nephropathic proteinuria in most people is later than the increase of these two cytokines. However, the rise of Th2 did not cause all patients to suffer from allergic diseases such as asthma, precisely because a type of Breg can protect patients from hyperactive autoimmune response even when exposed to allergens [155, 156]. And this kind of Breg is the Br1 cell.

## 5. Conclusion

We made three significant conclusions. First, we concluded that there are phenotypes and functions of Breg in humans. Second, IMN has many characteristics that are different from other autoimmune diseases. Third, we reasoned that the number of Br1 cells in the activated state in IMN patients is increased.

At present, there are abundant articles about the role of Breg in autoimmune diseases. However, its role in membranous nephropathy is still unknown. This is because it is widely believed that immune-regulating cells in the body play a role in alleviating or even blocking autoimmune diseases and are unlikely to become pathogenic. In this review, we discuss the main functions of Breg and propose relevant hypotheses based on the abnormal pathological and immunological characteristics of IMN. An interesting finding was that Breg has a cell that can secrete IgG4, and IgG4 is the main pathogenic antibody of IMN. Therefore, we believe that IMN may have abnormal Breg function and is related to the activity of Br1 cells. In addition, renal pathology in IMN patients, even at an early stage, is characterized by rare IgM deposition or CD8^+^ T cell infiltration. Therefore, we suspect that patients already have an autoimmune response at the beginning of IMN, and the number of Breg cells and Th2 cells in these patients is in a comparative advantage, which makes their autoimmune response mainly based on humoral immunity from the beginning. This is the central idea of our hypothesis.

Although Breg is only one type of B cell or immune regulatory cell, its role cannot be ignored. We hope to confirm these views through basic experiments and clinical trials, looking for the number of Breg and subtype abnormalities in the peripheral blood of IMN patients. When conditions permit, a case review is conducted in patients with IMN to investigate the changes of cytokines in their peripheral blood before and after the disease, so as to explore how these abnormal Breg proportions are activated. At the same time, in terms of treatment, we observed whether the efficacy of the drug was related to the change of Breg of the patient, and then developed a treatment method to prevent or inhibit the incidence of IMN and renal damage caused by IMN, so as to be beneficial to the clinical treatment of IMN patients. Finally, through the above series of studies, it is proved that Breg plays a crucial role in IMN, and some new Breg is even found to expand our understanding of Breg.

## Figures and Tables

**Table 1:** Breg found in human peripheral blood.

Name	Breg cell phenotype	Method of stimulation in vitro	Mechanism of suppression	References
B10	CD24^hi^CD27^+^	CD40L^+^CpG, LPS	IL-10	29
Immature B cell	CD24^hi^CD38^hi^CD27^−^	CD40L, CpG^+^pDC	IL-10, PD-L1, CD80/CD86	28
GrB^+^ B cell	CD38^+^CD1d^hi^IgM^+^CD147^+^	IL-21^+^ anti-BCR	IL-10, GrB, IDO	30
Tim-1 B cell	Tim-1^+^	—	IL-10, TGF-*β*	31
—	CD39^+^CD73^+^	CD40L^+^IL-4	Adenosine	64
—	IgD^lo^CD38^+^CD24^lo^CD27^−^	—	CD62L	65
Plasmablasts	CD24^hi^CD27^int^CD38^+^	GpG^+^IL-2^+^IL-6^+^IFN*α*	IL-10	32
Br1 cell	CD25^hi^CD71^hi^	CpG	IL-10, IgG4	33
B-1 cell	CD27^+^CD43^+^CD11b^+^	—	IL-10, CD80/CD86	34
